# SAINT-Angle: self-attention augmented inception-inside-inception network and transfer learning improve protein backbone torsion angle prediction

**DOI:** 10.1093/bioadv/vbad042

**Published:** 2023-04-05

**Authors:** A K M Mehedi Hasan, Ajmain Yasar Ahmed, Sazan Mahbub, M Saifur Rahman, Md Shamsuzzoha Bayzid

**Affiliations:** Department of Computer Science and Engineering, Bangladesh University of Engineering and Technology, Dhaka 1205, Bangladesh; Department of Computer Science and Engineering, Bangladesh University of Engineering and Technology, Dhaka 1205, Bangladesh; Department of Computer Science and Engineering, Bangladesh University of Engineering and Technology, Dhaka 1205, Bangladesh; Department of Computer Science, University of Maryland, College Park, MD 20742, USA; Department of Computer Science and Engineering, Bangladesh University of Engineering and Technology, Dhaka 1205, Bangladesh; Department of Computer Science and Engineering, Bangladesh University of Engineering and Technology, Dhaka 1205, Bangladesh

## Abstract

**Motivation:**

Protein structure provides insight into how proteins interact with one another as well as their functions in living organisms. Protein backbone torsion angles (ϕ and ψ) prediction is a key sub-problem in predicting protein structures. However, reliable determination of backbone torsion angles using conventional experimental methods is slow and expensive. Therefore, considerable effort is being put into developing computational methods for predicting backbone angles.

**Results:**

We present SAINT-Angle, a highly accurate method for predicting protein backbone torsion angles using a self-attention-based deep learning network called SAINT, which was previously developed for the protein secondary structure prediction. We extended and improved the existing SAINT architecture as well as used transfer learning to predict backbone angles. We compared the performance of SAINT-Angle with the state-of-the-art methods through an extensive evaluation study on a collection of benchmark datasets, namely, TEST2016, TEST2018, TEST2020-HQ, CAMEO and CASP. The experimental results suggest that our proposed self-attention-based network, together with transfer learning, has achieved notable improvements over the best alternate methods.

**Availability and implementation:**

SAINT-Angle is freely available as an open-source project at https://github.com/bayzidlab/SAINT-Angle.

**Supplementary information:**

Supplementary data are available at *Bioinformatics Advances* online.

## 1 Introduction

Proteins are responsible for various functions in cells and their functions are usually determined by their 3D structures. However, the experimental determination of protein structures using X-ray crystallography, cryogenic electron microscopy (cryo-EM) and nuclear magnetic resonance spectroscopy is costly and time- and labour-intensive (Jiang *et al.*, 2017). Therefore, developing efficient computational approaches for determining protein structures has been gaining increasing attention from the scientific community (AlQuraishi, 2019; Greener *et al.*, 2019; Senior *et al.*, 2020; Xu, 2019; Xu *et al.*, 2020). The backbone torsion angles [the measurements of the residue-wise torsion (Ramachandran *et al.*, 1963)] play a critical role in protein structure prediction and investigating protein folding (Adhikari *et al.*, 2012; Gao *et al.*, 2018; Tian *et al.*, 2020). Therefore, protein structure prediction is often divided into smaller and more doable sub-problems (Heffernan *et al.*, 2017) such as backbone torsion angles prediction. As a result, accurate prediction of torsion angles can significantly advance our understanding of the 3D structures of proteins.

Given the growing availability of protein databases and rapid advances in machine learning (ML) methods (especially, the deep learning techniques), application of ML techniques to leverage the available data in accurate prediction of backbone angles has gained significant attention.

Earlier ML-based methods used neural network (Wu and Zhang, 2008), support vector machine (SVM) (Wu and Zhang, 2008) and hidden Markov model (HMM) (Bystroff *et al.*, 2000; Karchin *et al.*, 2003) to predict discrete states of torsion angles ϕ and ψ. Real-SPINE (Dor and Zhou, 2007) leveraged an integrated system of neural networks to predict the real values of dihedral angles.

Several deep learning-based techniques have recently been developed that can predict backbone torsion angles with a reasonable accuracy. SPIDER2 (Heffernan *et al.*, 2015) used iterative neural network to predict the backbone torsion angles, while SPIDER3 (Heffernan *et al.*, 2017) leveraged the bidirectional recurrent neural network (BiRNN) (Schuster and Paliwal, 1997) to capture the long-range interactions among amino acid residues in a protein molecule. MUFOLD (Fang *et al.*, 2018b) used deep residual inception models (Szegedy *et al.*, 2017) to measure the short-range and long-range interactions among different amino acid residues. Similar to SPIDER3, NetSurfP-2.0 (Klausen *et al.*, 2019) used the bidirectional recurrent neural network to capture the long-range interactions. Some studies also emphasized on input feature selection. RaptorX-Angle (Gao *et al.*, 2018) took advantage of both discrete and continuous representation of the backbone torsion angles and explored the efficacy of different types of features, such as position-specific scoring matrix (PSSM) using PSI-BLAST (Altschul *et al.*, 1997) and position-specific frequency matrix (PSFM) using HHpred (Remmert *et al.*, 2012; Söding, 2005).

SPOT-1D (Hanson *et al.*, 2019) is an ensemble of nine base models based on the architecture of long short-term memory (LSTM) (Hochreiter and Schmidhuber, 1997), bidirectional recurrent neural network (BiRNN) (Schuster and Paliwal, 1997) and deep residual network (ResNet) (He *et al.*, 2016). They leveraged the predicted contact-map information produced by SPOT-Contact (Hanson *et al.*, 2018) to improve the performance. OPUS-TASS (Xu *et al.*, 2020) is another state-of-the-art method, which is an ensemble of 11 base models based on convolutional neural network (CNN) modules (LeCun *et al.*, 1998), bidirectional long short-term memory (BiLSTM) modules (Hochreiter and Schmidhuber, 1997) and modified Transformer modules (Vaswani *et al.*, 2017). OPUS-TASS is a multi-task learning model (Lounici *et al.*, 2009), maximizing the generalization of neural network, in which the same network was trained for six different prediction tasks. Accuracy of the backbone torsion angles prediction does not rely only on the architecture used in deep learning-based methods but also on the input features extracted from protein sequences. PSSM profiles, HMM profiles (Remmert *et al.*, 2012), physicochemical properties (PP) (Meiler *et al.*, 2001) and amino acid (AA) labels of the residues in proteins are widely used features in predicting protein properties. Recently, the authors of ESIDEN (Xu *et al.*, 2021) introduced four evolutionary signatures as novel features, namely relative entropy (RE), degree of conservation (DC), position-specific substitution probabilities (PSSP) and Ramachandran basin potential (RBP). ESIDEN is an evolutionary signatures-driven deep neural network developed based on the architecture of long short-term memory (LSTM) and bidirectional long short-term memory (BiLSTM) and achieved notable improvements over other alternate methods. Protein language models, motivated by natural language processing (NLP), have lately been introduced to extract features from protein primary sequence for downstream analyses. The authors of SPOT-1D-LM (Singh et al., 2022b) explored two of the contemporary protein language models ProtTrans (Elnaggar *et al*., 2021) and ESM-1b (Rao *et al.*, 2021) to extract features from protein sequence and developed a method to predict 1D structural properties of proteins.

In this study, we present SAINT-Angle, a highly accurate method for protein backbone torsion angle prediction, which is built on our previously proposed architecture of self-attention augmented inception-inside-inception network (SAINT) (Uddin *et al.*, 2020) for protein secondary structure (SS) prediction. We adapted the SAINT architecture for torsion angle prediction and further augmented the basic architecture of SAINT by incorporating the deep residual network (He *et al.*, 2016). We present a successful utilization of transfer-learning from pretrained transformer-like models by ProtTrans (Elnaggar *et al.*, 2021) in backbone angle prediction. SAINT-Angle is capable of capturing both short- and long-range interactions among amino acid residues. SAINT-Angle was compared with the best alternate methods on a collection of widely used benchmark datasets, namely TEST2016 (Hanson *et al.*, 2018), TEST2018 (Hanson *et al.*, 2019), TEST2020-HQ (Singh et al., 2022b), CAMEO (Xu *et al.*, 2021) and CASP (Uddin *et al.*, 2020; Xu *et al.*, 2021). SAINT-Angle significantly outperformed other competing methods and achieved the best known ϕ and ψ prediction accuracy.

## 2 Approach

### 2.1 Feature representation

SAINT-Angle takes a protein sequence feature vector $X=\{x_{1},x_{2},\ldots ,x_{i},x_{i+1}\ldots ..,x_{N}\}$ as input, where $x_{i}$ is the vector corresponding to the *i*th residue of that protein. For each of the residue, SAINT-Angle has four regression nodes which predict sin(ϕ), cos(ϕ), sin(ψ) and cos(ψ), respectively. SAINT-Angle uses three different sets of features which we call (i) *Base features*, (ii) *ProtTrans features* and (iii) *Window features*.

The *Base feature* class consists of a feature vector of length 57 for each residue. It contains features from PSSM profiles, HMM profiles and physicochemical properties (PCP) (Meiler *et al.*, 2001). We ran PSI-BLAST (Altschul *et al.*, 1997) against the *Uniref*90 (UniProt Consortium, 2007) database with an inclusion threshold of 0.001 and 3 iterations to generate PSSM profiles. We used HHblits (Remmert *et al.*, 2012) using the default parameters against the *uniprot20_2013_03* sequence database to generate the HMM profiles. HHblits also generates seven transition probabilities and three local alignment diversity values, which we used as features as well. Seven physicochemical properties of each amino acid [steric parameters (graph-shape index), polarizability, normalized van der Waals volume, hydrophobicity, isoelectric point, helix probability and sheet probability] were obtained from Meiler *et al.* (2001). Thus, the dimension of our base feature class for each residue is 57 as this is the concatenation of 20 features from PSSM, 30 features from HMM and 7 features from physicochemical properties.

The *ProtTrans features*, generated by the pretrained language model for proteins developed by Elnaggar *et al.* (2021), consist of a feature vector of length 1024 for each residue. Elnaggar *et al.* (2021) trained two auto regression language models [Transformer-XL (Dai *et al.*, 2019) and XLNet (Yang *et al.*, 2019)] on data containing up to 393 billion amino acids from 2.1 billion protein sequences in a self-supervised manner, considering each residue as a ‘word’ [similar to language modeling in natural language processing (Devlin et al., 2019)]. Features extracted from ProtT5-XL-UniRef50 language model were used in our experiments because, in general, these features from protein language models, including ProtTrans and ESM-1b (Rao *et al.*, 2021), were shown to contribute to improving the performance of methods on residue-level prediction tasks (Mahbub and Bayzid, 2022; Singh et al., 2022a,b). Other suitable protein language models, apart from ProtT5-XL-UniRef50, may also be used because our proposed architecture is agnostic about these language models. ProtT5-XL-UniRef50 language model generates a sequence of embedding vectors $q=\{q_{1},q_{2},q_{3},\ldots ,q_{N}\}$, $q_{i}\in R^{d_{\text{prottrans}}}\;(d_{\text{prottrans}}=1024)$ for each residue $X_{i}$.

*Window features* are generated by windowing the predicted contact information as was done in SPOT-1D and was subsequently used in SAINT. We used SPOT-Contact to generate the contact-maps. We varied the window lengths (the number of preceding or succeeding residues whose pairwise contact information were extracted for a target residue) to generate different dimensional features. We used three different window lengths (10,20,50) to generate the window features, and denote them by Win10, Win20 and Win50, respectively.

### 2.2 Architecture of SAINT-Angle

The architecture of SAINT-Angle can be split into three separate discussions: (i) the architecture of SAINT (Uddin *et al.*, 2020), which was proposed for protein secondary structure prediction and our proposed modifications, (ii) the base model architectures of SAINT-Angle that have been applied in the ensemble and (iii) the overall pipeline of SAINT-Angle.

#### 2.2.1 Architecture of SAINT

We discuss the architecture of SAINT briefly here so as to make this article self-contained and easy to follow. For details of SAINT, we refer the reader to Uddin *et al.* (2020). We also discuss the modifications that we have made to the original SAINT architecture to make it suitable for the task of backbone torsion angle prediction. Two of the core components of SAINT are: (i) the self-attention module, and (ii) 2A3I module, which will be discussed in subsequent sections.

##### 2.2.1.1 Self-attention module

**Fig. 1.**
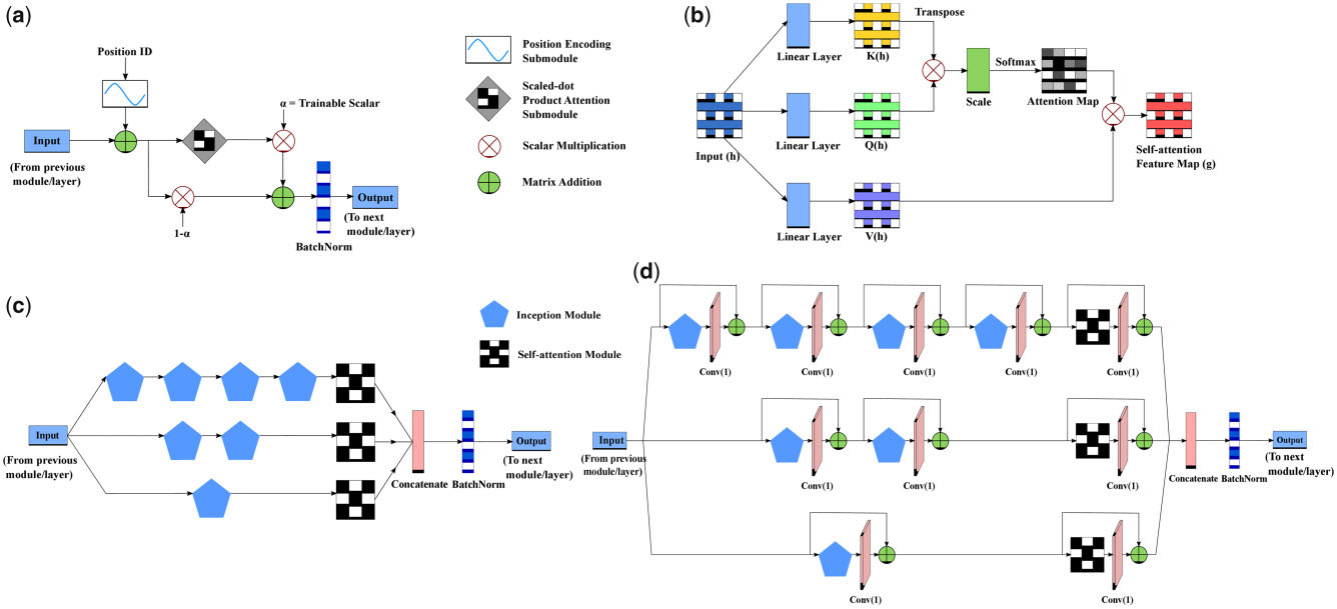
Schematic diagrams of various components of the SAINT-Angle architecture. (**a**) Self-attention module, (**b**) scaled-dot product attention sub-module (Vaswani *et al.*, 2017), (**c**) 2A3I module proposed by Uddin *et al.* (2020) and (**d**) RES-2A3I module which augments the 2A3I module with residual connections. Conv(x) denotes a 1-D convolution layer with a kernel of size *x*

The self-attention module, as shown in Figure 1a, that we designed and augmented with the Deep3I network (Fang *et al.*, 2018a) is inspired by the self-attention module developed by Vaswani *et al.* (2017). We pass two inputs to our self-attention module: (i) the features from the previous inception module or layer, $x\;\in R^{d_{\text{protein}}\times d_{\text{feature}}}$, and (ii) position identifiers, $\mathrm{pos}\_id\;\in R^{d_{\text{protein}}}$, where $d_{\text{protein}}$ is the length of the protein sequence, and $d_{\text{feature}}$ is the length of the feature vector.

##### 2.2.1.2 Positional encoding sub-module

As the relative or absolute positions of the residues in a protein sequence are important, we need to provide this positional information in our model as shown in Figure 1a. The *Positional Encoding* $\mathrm{PosEn}c_{p}$ for a position *p* can be defined as follows (Vaswani *et al.*, 2017). where *i* is the dimension. The above-mentioned function allows the model to learn to attend by relative positions. The inputs *x* is added to the output of positional encoding, resulting in a new representation *h* (Eqn. 3). This new representation *h* not only contains the information extracted by the previous layers or modules but also the information about individual positions.


(1) $$\mathrm{PosEn}c_{\left(p,2i\right)}=\text{sin}(p/10\;000^{2i/d_{\text{feature}}})$$

(2) $$\mathrm{PosEn}c_{\left(p,2i+1\right)}=\text{cos}(p/10\;000^{2i/d_{\text{feature}}}),$$

(3) $$h_{\text{pos}}=x_{\text{pos}}+\mathrm{PosEn}c_{\text{pos}}$$

##### 2.2.1.3 Scaled dot-product attention sub-module

We provide the output of the positional encoding sub-module $h\in R^{d_{\text{protein}}\times d_{\text{feature}}}$ as the input to the *scaled-dot product* attention sub-module as shown in Figure 1b. This input vector is first transformed into three feature spaces *Q*, *K*, *V*, representing query, key and value, respectively. We use three learnable parameter matrices $W_{Q}$, $W_{K}$, $W_{V}$ for this transformation such that $Q(h)=W_{Q}h$, $K(h)=W_{K}h$, $V(h)=W_{V}h$. We then compute the scaled dot-product $s_{i,j}$ of two vectors $h_{i}$ and $h_{j}$ using Q(h) and K(h) vectors. This scaled dot-product $s_{i,j}$ is subsequently used to compute the attention weights $e_{j,i}$ ($e\;\in R^{d_{\text{protein}}\times d_{\text{feature}}}$), representing how much attention to provide to the vector *i* while synthesizing the vector at position *j*. The output of the scaled dot-product attention sub module *g* is then computed by multiplying the value vector V(h) with the previously calculated attention weights *e* and subsequently applying batch normalization (Ioffe and Szegedy, 2015) to reduce the internal covariate shift (Eqn. 4). Please see Uddin *et al.* (2020) for details.


(4) $$g_{j}=\mathrm{BatchNorm}(\sum_{n=1}^{d_{\text{protein}}}e_{j,i}V(h_{i}))$$

##### 2.2.1.4 2A3I and RES-2A3I modules

Fang *et al.* (2018a) used an assembly of inception modules, which they call 3I (Inception-Inside-Inception) module, in their proposed method MUFOLD-SS to predict protein SS. Uddin *et al.* (2020) augmented this with attention modules in order to effectively capture both short- and long-range interactions by placing the self-attention modules (described in Sect. 2.2.1) in each branch of the 3I module as shown in Figure 1c. This is called the 2A3I (attention augmented inception-inside-inception) module. In this study, we further extended this module by placing residual connections in each of the inception and self-attention modules (Fig. 1d). Residual connections (He *et al.*, 2016) tackle *vanishing gradient problem* (Bengio *et al.*, 1994) and help make our model more stable. Weight gradients in a neural network are typically very small. During the training of a deep neural network, these small gradients are multiplied by additional small values, resulting in a very small gradient in the earlier layers, and sometimes little or no gradient update at all (as useful gradient information cannot be propagated from the output end of the model back to the layers). This vanishing gradient problem can be addressed by residual connections, producing a more noise stable model with improved learning capacity (Yu and Tomasi, 2019). We call this residual connection-augmented module the *RES-2A3I* module.

#### 2.2.2 Base models of SAINT-Angle

We developed the following three architectures that we utilize in an ensemble network to create: (i) *Basic* architecture, (ii) *ProtTrans* architecture and (iii) *Residual* architecture.

##### 2.2.2.1 Basic architecture

**Fig. 2.**
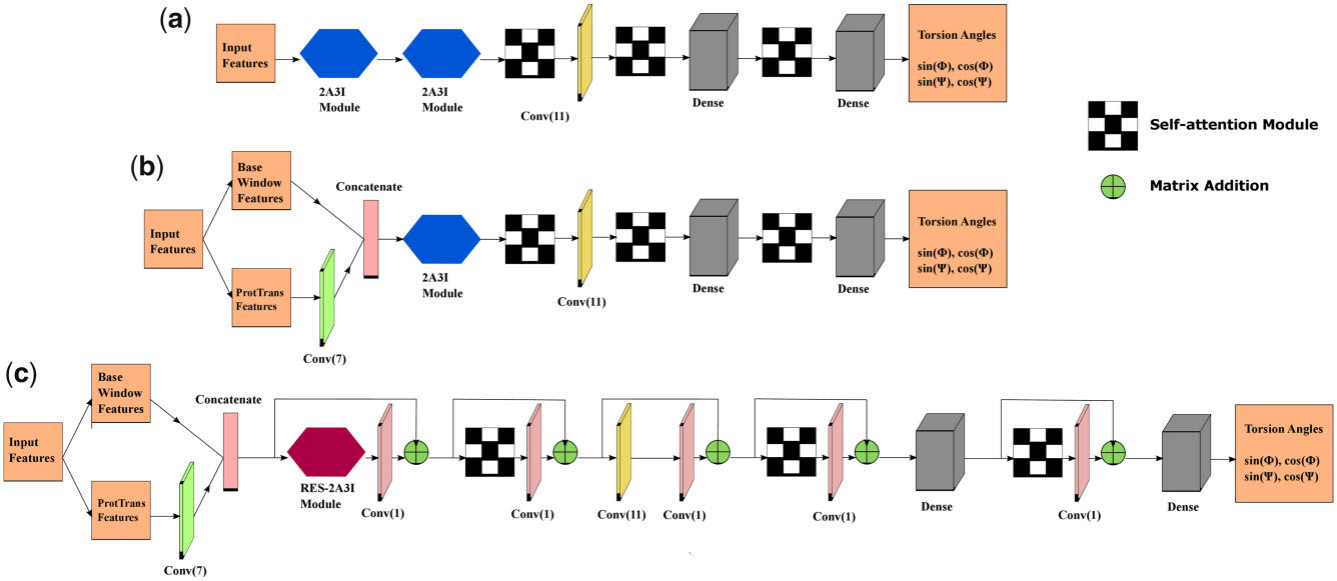
Schematic diagrams of the architectures of the base models used in SAINT-Angle. (**a**) The *Basic* architecture which was proposed by Uddin *et al.* (2020), (**b**) the *ProtTrans* architecture and (**c**) the *Residual* architecture which augments the *ProtTrans* architecture with residual connections

Figure 2a shows the schematic diagram of our *Basic* architecture which is identical to the original SAINT architecture proposed for protein SS prediction (Uddin *et al.*, 2020). It starts with two consecutive 2A3I modules followed by a self-attention module. This self-attention module supplements the amount of non-local interactions that have been captured by previous two 2A3I modules. Next, we have an 1D convolutional layer with window size 11. The output of the convolutional layer is passed through another self-attention module, followed by two dense layers, with yet another self-attention module placed in between these two dense layers. This self-attention module helps in understanding how the residues align and interact, making it easier to comprehend the behavior of the model. The final dense layer has four regression nodes that infer sin(ϕ), cos(ϕ), sin(ψ) and cos(ψ).

##### 2.2.2.2 ProtTrans architecture

We developed the *ProtTrans* architecture (Fig. 2b) to effectively use the ProtTrans features by treating them differently from the base and window features. We pass the ProtTrans features to a 1D convolution layer with kernel size 7. This convolutional layer acts as a local feature extractor, capturing local interactions between residues and reducing the dimension of the ProtTrans features from a 1024D feature vector to a 300D feature vector, allowing the model to filter out less important information. It also aids in avoiding over-fitting and reducing the number of trainable parameters. The output of this 1D convolution layer is then concatenated with the base and window features. The concatenated vector is then passed through a single 2A3I module. Note that, unlike the basic SAINT architecture, we have only one 2A3I module as we observed that two 2A3I modules do not provide notable advantage in this architecture but increase the training time. The rest of the architecture is similar to the basic SAINT architecture.

##### 2.2.2.3 Residual architecture

The *Residual* architecture (Fig. 2c) is similar to the *ProtTrans* architecture except for two differences: (i) we have added residual connections (He *et al.*, 2016) between different components as shown in Figure 2c, and (ii) we have used the RES-2A3I module instead of the 2A3I module. Residual connections enable the deeper layers to use the features extracted from the earlier layers. Usually, the deeper level layers use features that are highly convoluted and lower in resolution. Residual connections help the deeper layers leverage the low-level and high dimensional features. It also helps to make the model stable.

#### 2.2.3 Overview of SAINT-Angle

*SAINT-Angle* is an ensemble network of eight models with different combinations of architectures (discussed in Sect. 2.2.2) and features (discussed in Sect. 2.1)—resulting in a set of diverse learning paths leveraging different types of features. Table 1 shows the architectures and features used in these eight models. Details of the ensemble and the individual models therein are presented in Supplementary Material. We used the same training and validation sets that were used by SPOT-1D to train these models and tune necessary hyperparameters.

**Table 1.** Eight models used in SAINT-angle

Model	Architecture	Feature set
1	Basic	Base
2	Basic	Base+Win10
3	ProtTrans	Base+ProtTrans
4	ProtTrans	Base+ProtTrans+Win10
5	ProtTrans	Base+ProtTrans+Win20
6	ProtTrans	Base+ProtTrans+Win50
7	Residual	Base+ProtTrans
8	Residual	Base+ProtTrans+Win10

*Note*: We show the architectures and features used in these eight models.

Each model was trained using Adam optimizer (Kingma and Ba, 2015) with an initial learning rate of 0.0001 which was subsequently reduced by half when the accuracy of the validation set did not improve for five consecutive epochs.

We performed an extensive ablation study to assess the contribution of different feature sets and model architectures used in SAINT-Angle (see Supplementary Sect. S3 in Supplementary Material). The ablation studies demonstrate the motivation behind using different features and different model architectures that are tailored for particular feature sets.

## 3 Results and discussion

We performed an extensive evaluation study, comparing SAINT-Angle with the recent state-of-the-art methods on a collection of widely used benchmark dataset.

### 3.1 Dataset

#### 3.1.1 Training and validation dataset

The SPOT-1D dataset (Hanson *et al.*, 2018, 2019) was used for training and validation of SAINT-Angle. These proteins were culled from the PISCES server (Wang and Dunbrack, 2003) on February 2017 with resolution <2.5 Å, R-free<1, and a sequence identity cutoff of 25% according to BlastClust (Altschul *et al.*, 1997). The proteins consisting of over 700 amino acid residues were removed by the authors of SPOT-1D to fit in the SPOT-Contact (Hanson *et al.*, 2018) pipeline. As a result, 10 029 and 983 proteins remained in the training and validation sets, respectively.

#### 3.1.2 Test dataset

We assessed the performance of SAINT-Angle and other competing methods on a collection of widely used test sets, which are briefly described in Supplementary Sect. 4 in the Supplementary Material.

### 3.2 Performance evaluation

We compared our proposed SAINT-Angle with several state-of-the-art methods: SPOT-1D-LM, SPOT-1D-Single (Singh *et al.*, 2021), OPUS-TASS, SPOT-1D, NetSurfP-2.0, MUFOLD, SPIDER3 and RaptorX-Angle. We also compared SAINT-Angle with a recent and highly accurate method ESIDEN (Xu *et al.*, 2021). We evaluated the performance of backbone torsion angles prediction methods using mean absolute error (MAE) which is the measure of average absolute difference between native values (*T*) and predicted values (*P*) over all amino acid residues in a protein. The minimum value between |T−P| and $360^{{}^{\circ}}-|T-P|$ was taken in order to reduce the periodicity of an angle (Xu *et al.*, 2021) as given in Eqn. 5. Here *N* is the number of proteins, $L_{i}$ is the total number of amino acid residues in the *i*th protein. $T_{ij}$ and $P_{ij}$ are the values of native and predicted angles of the *j*th amino acid residue in the *i*th protein, respectively. We performed Wilcoxon signed-rank test (Wilcoxon *et al.*, 1970) (with α=0.05) to measure the statistical significance of the differences between two methods.


(5) $$\text{MAE}=\frac{1}{\sum_{i=1}^{N}L_{i}}\sum_{i=1}^{N}\sum_{j=1}^{L_{i}}\text{min}(|T_{ij}-P_{ij}|,\;360^{{}^{\circ}}-|T_{ij}-P_{ij}|)$$

### 3.3 Results on benchmark datasets

The comparison of SAINT-Angle with other state-of-the-art methods on TEST2016 and TEST2018 is shown in Table 2. Experimental results show that SAINT-Angle outperforms all other methods on both TEST2016 and TEST2018 datasets except that SPOT-1D-LM is slightly better than SAINT-Angle in ϕ angle prediction on TEST2018 dataset. Especially, the improvement in MAE(ψ) is substantial—almost two degrees over the third best method OPUS-TASS, and more than 2–6 degrees compared to other methods. Notably, even the individual eight base models used in SAINT-Angle achieve comparable or better performance than most other methods (see Supplementary Table S4 in Supplementary Material). Among these individual base models, the improvements of the models with *ProtTrans* architecture and ProtTrans features (Models 3, 4, 5 and 6 in Table 1) over the *Basic* architecture with base features (Models 1 and 2) are notable—indicating the successful utilization of ProtTrans-based transfer learning. Statistical tests suggests that these improvements of SAINT-Angle over other methods are statistically significant (*P*-value ≪0.05). RaptorX-Angle performed poorly compared to other methods on both these datasets. OPUS-TASS and SPOT-1D produced similar results, with OPUS-TASS obtaining marginally better results than SPOT-1D. Both OPUS-TASS and SPOT-1D obtained notably better results than NetSurfP, MUFOLD and SPIDER3.

**Table 2.** Performance [in terms of MAE(ϕ) and MAE(ψ)] of SAINT-Angle and other state-of-the-art methods on TEST2016 and TEST2018

Method	TEST2016		TEST2018	
	MAE(ϕ)	MAE(ψ)	MAE(ϕ)	MAE(ψ)
RaptorX-Angle^a^	18.08	26.68	21.01	35.95
SPIDER3^a^	17.88	26.66	18.38	28.10
MUFOLD^a^	–	–	17.78	27.24
NetSurfP-2.0^a^	–	–	17.90	26.63
SPOT-1D^a^	16.27	23.26	16.89	24.87
OPUS-TASS^b^	*15.78*	*22.46*	16.40	24.06
SPOT-1D-LM^c^	–	–	**15.99**	*23.74*
SAINT-Angle	**15.45**	**20.77**	*16.25*	**22.37**

*Note*: The best and the second best results are shown in bold and italic, respectively. Values which were not reported by the corresponding source are indicated by ‘–’.

a Results reported by SPOT-1D (Hanson *et al.*, 2019).

b Results reported by OPUS-TASS (Xu *et al.*, 2020).

c Results reported by SPOT-1D-LM (Singh et al., 2022b).

The performance of SAINT-Angle and other competing methods on three CASP datasets (CASP12, CASP13 and CASP-FM) is shown in Table 3. SAINT-Angle consistently outperformed other methods on these datasets, with only one exception on CASP-FM where OPUS-TASS obtained a better MAE(ϕ) than SAINT-Angle albeit the difference is very small (0.01 degree). Similar to TEST2016 and TEST2018 datasets, the improvements of SAINT-Angle over other methods in MAE(ψ) are more substantial than those in MAE(ϕ). The improvements of SAINT-Angle over other methods are statistically significant (*P*-value ≪0.05).

**Table 3.** Performance [in terms of MAE(ϕ) and MAE(ψ)] of SAINT-Angle and other state-of-the-art methods on three CASP datasets

Method	CASP12 (55)		CASP13 (32)		CASP-FM (56)	
	MAE					
	**(** ϕ **)**	**(** ψ **)**	**(** ϕ **)**	**(** ψ **)**	**(** ϕ **)**	**(** ψ **)**
SPOT-1D^a^	18.44	26.90	18.48	26.73	19.39	30.10
OPUS-TASS^a^	*18.08*	*25.98*	*17.89*	*25.93*	**18.85**	*28.00*
SAINT-Angle	**17.91**	**24.76**	**17.55**	**23.96**	*18.86*	**27.65**

*Note*: The best and the second best results are shown in bold and italic, respectively.

a Results reported in OPUS-TASS.

### 3.4 Results on TEST2020-HQ dataset

We further compared the backbone torsion angles prediction performance of SAINT-Angle with the best existing methods on a newly introduced dataset TEST2020-HQ dataset, which was previously assembled and analyzed by SPOT-1D-LM (Singh et al., 2022b). Given the difficulty in generating window features, we excluded certain base models, from the eight models as listed in Table 1, that required window features for prediction. Thus, we used an ensemble of only three base models (Models 1, 3 and 7).

The performance comparison of SAINT-Angle with other methods is shown in Table 4. SAINT-Angle outperformed other methods by a large margin (even with an ensemble of three models with base and ProtTrans features). This further supports the superiority of our model architectures over other methods.

**Table 4.** Performance of SAINT-Angle (ensemble of three base models without window features), NetSurfP-2.0, SPOT-1D, SPOT-1D-Single and SPOT-1D-LM on TEST2020-HQ dataset

Method	TEST2020-HQ	
	MAE(ϕ)	MAE(ψ)
NetSurfP-2.0^a^	19.90	31.29
SPOT-1D^a^	*18.78*	*28.87*
SPOT-1D-Single^a^	23.01	42.62
SPOT-1D-LM^a^	19.52	32.01
SAINT-Angle	**17.94**	**26.90**

*Note*: The best and the second best results are shown in bold and italic, respectively.

a Results reported by SPOT-1D-LM.

### 3.5 Comparison of SAINT-Angle with ESIDEN

ESIDEN (Xu *et al.*, 2021)—a recent, highly accurate recurrent neural network-based method—introduced and leveraged four evolutionary signatures as novel features, namely relative entropy (RE), degree of conservation (DC), position-specific substitution probabilities (PSSP) and Ramachandran basin potential (RBP). They showed that these novel features, along with classical features such as PSSM, physicochemical properties (PP) and amino acid (AA), result in significant improvements in protein torsion angle prediction. As ESIDEN is not an ensemble-based network, in order to make a fair comparison with ESIDEN and to further assess the efficacy of the novel evolutionary features, we trained our basic architecture (discussed in Sect. 2.2.2.1) using the features used by ESIDEN and evaluated its performance on a collection of datasets compiled and analyzed by the authors of ESIDEN. This will enable us to assess the performance of the basic SAINT architecture using the features used by ESIDEN (i.e. without the ProtTrans-based transfer learning and the ensemble network). We call this *Basic* architecture with ESIDEN features *SAINT-Angle-Single*. We obtained the evolutionary features for the SPOT-1D training dataset and a collection of test datasets from the authors of ESIDEN. We also trained our ensemble network using the base features along with the novel ESIDEN features [i.e. 20 types of amino acids (AA) and four evolutionary features DC, RE, PSSP and RBP]. The evolutionary features of ESIDEN were shown to be reasonably powerful (Xu *et al.*, 2021), which has been further supported by our experimental results as well (discussed later in this section). On the other hand, the window features are difficult to compute (Xu *et al.*, 2020). Therefore, in these experiments, we did not use the window features to keep the dimension of the feature vector manageable as well as to best take advantage of the evolutionary features used in ESIDEN. Thus, after removing the window features when ESIDEN features are available, we had three models (out of eight models listed in Table 1) to use for the ensemble network (see Supplementary Sect. 2.1 and Supplementary Table S5 in Supplementary Material). In order to distinguish this ensemble of three base models using the ESIDEN features from the ensemble of eight models, we call this SAINT-Angle${}^{*}$.

The comparison of SAINT-Angle-Single, SAINT-Angle${}^{*}$ (ensemble of three models using the ESIDEN features) and SAINT-Angle (ensemble of eight models without ESIDEN features) with ESIDEN on TEST2016 and TEST2018 datasets is shown in Table 5. ESIDEN is notably better than the SPOT-1D, OPUS-TASS, SPOT-1D-LM as well as SAINT-Angle, especially for predicting the ψ angle [around $4^{{}^{\circ}}$ improvement in MAE(ψ)]. Note however that SAINT-Angle, unlike ESIDEN, does not use the four evolutionary features. Interestingly, SAINT-Angle-Single, which leverages ESIDEN features, is remarkably better than ESIDEN [$∼\;2^{{}^{\circ}}$ improvement in MAE(ψ)] as well as other methods. This shows the superiority of our SAINT architecture over the ESIDEN architecture.

**Table 5.** Performance of SAINT-Angle-Single (basic SAINT architecture with base and ESIDEN features), SAINT-Angle${}^{*}$ (ensemble of three models using the ESIDEN features) and SAINT-Angle (ensemble of eight models without ESIDEN features), and ESIDEN on TEST2016 and TEST2018

Method	TEST2016		TEST2018	
	MAE(ϕ)	MAE(ψ)	MAE(ϕ)	MAE(ψ)
SPOT-1D	16.27	23.26	16.89	24.87
OPUS-TASS	15.78	22.46	16.40	24.06
SPOT-1D-LM	–	–	15.99	23.74
ESIDEN^a^	15.48	19.25	16.00	20.28
SAINT-Angle	15.45	20.77	16.25	22.37
SAINT-Angle-Single	*14.39*	*17.03*	**14.89**	**17.94**
SAINT-Angle${}^{*}$	**14.18**	**16.59**	*15.48*	*18.77*

*Note*: The best and the second best results are shown in bold and italic, respectively.

a Results reported by ESIDEN (Xu *et al.*, 2021).

The performance of SAINT-Angle-Single and SAINT-Angle${}^{*}$ is mixed on these two datasets. SAINT-Angle${}^{*}$ is better than SAINT-Angle-Single on TEST2016 dataset whereas SAINT-Angle-Single is better than SAINT-Angle${}^{*}$ on TEST2018. Remarkably, both of them achieved substantial improvements over ESIDEN. Moreover, both SAINT-Angle-Single and SAINT-Angle${}^{*}$ outperformed SAINT-Angle—showing the power of the evolutionary features proposed by ESIDEN. The improvements of SAINT-Angle-Single and SAINT-Angle${}^{*}$ over ESIDEN and SAINT-Angle are statistically significant (P-value≪0.05).

We further assessed the performance of SAINT-Angle-Single in comparison with ESIDEN and other methods on five other benchmark datasets that were compiled and used by the authors of ESIDEN, namely CAMEO109 and four CASP datasets (CASP11, CASP12, CASP13, CASP14). Note that these CASP datasets [analyzed in ESIDEN (Xu *et al.*, 2021)] are different from the CASP datasets in Table 3 (which was used by SAINT). Results on the CAMEO109 dataset are shown in Table 6. ESIDEN is better than other existing methods in terms of MAE(ψ) ($∼1^{{}^{\circ}}$ improvement), but SPOT-1D obtained slightly better MAE(ϕ) than ESIDEN. SAINT-Angle-Single outperformed ESIDEN and other methods in terms of both MAE(ϕ) and MAE(ψ). Especially, it obtained around two degrees of improvement over ESIDEN in MAE(ψ).

**Table 6.** Performance of SAINT-Angle-Single and other state-of-the-art methods on CAMEO109 and four CASP datasets

Method	CAMEO109		CASP11 (27)		CASP12 (11)		CASP13 (13)		CASP14 (8)	
	MAE(ϕ)	MAE(ψ)	MAE(ϕ)	MAE(ψ)	MAE(ϕ)	MAE(ψ)	MAE(ϕ)	MAE(ψ)	MAE(ϕ)	MAE(ψ)
RaptorX-Angle^a^	19.57	33.96	20.33	40.05	21.71	38.22	22.73	41.18	24.95	48.06
SPIDER3^a^	17.89	28.32	19.19	34.63	21.14	34.92	*22.48*	38.46	23.41	38.79
SPOT-1D^a^	*16.49*	25.17	18.54	25.77	20.21	31.71	22.60	34.28	23.42	33.44
ESIDEN^a^	16.57	*24.25*	**17.25**	*23.30*	*19.94*	*28.86*	**22.15**	*32.40*	*23.01*	*29.96*
SAINT-Angle-Single	**16.40**	**22.77**	*17.40*	**22.52**	**19.58**	**27.11**	**22.15**	**31.46**	**22.67**	**29.83**

*Notes:* The numbers of proteins in these datasets are shown in parentheses. The best and the second best results are shown in bold and italic, respectively.

a Results reported by ESIDEN.

Results on four CASP datasets are shown in Table 6. SAINT-Angle-Single and ESIDEN are significantly better than other existing methods, especially for ψ where ESIDEN and SAINT-Angle achieved more than $∼10^{{}^{\circ}}$ improvements over other methods. Remarkably, SAINT-Angle-Single outperformed all other methods (including ESIDEN) across all the datasets in terms of both MAE(ϕ) and MAE(ψ), with only one exception where ESIDEN obtained a better MAE(ϕ) than SAINT-Angle-Single on the CASP11 dataset.

### 3.6 Analysis of the predicted angles

We further investigated the predicted protein backbone torsion angles from SAINT-Angle and other contemporary methods to obtain better insights on the performances of various methods.

#### 3.6.1 Impact of long-range interactions

**Fig. 3.**
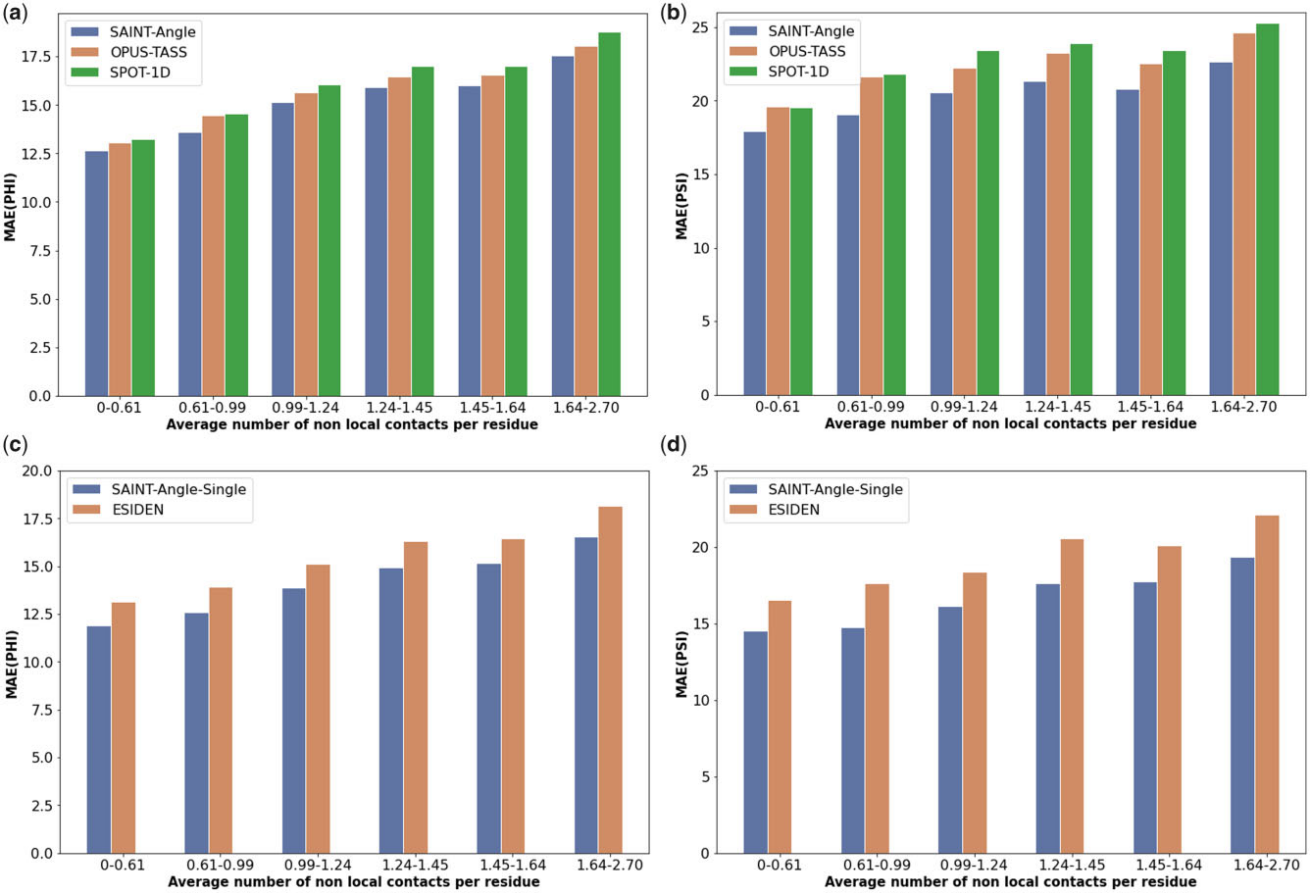
MAE(ϕ) and MAE(ϕ) of SAINT-Angle and the best alternative methods under various levels of non-local interactions. We show the results on the TEST2016 test set using six bins of proteins. (**a**) MAE(ϕ) of SAINT-Angle, OPUS-TASS and SPOT-1D under various levels of non-local contacts. (**b**) MAE(ψ) of SAINT-Angle, OPUS-TASS and SPOT-1D under various levels of non-local contacts. (**c**–**d**) MAE(ϕ) and MAE(ψ) of SAINT-Angle-Single and ESIDEN under various levels of non-local contacts

We investigated the effect of long-range interactions among amino acid residues in protein torsion angle prediction. Two residues at sequence position *i* and *j* are considered to have non-local contact or interaction if they are at least twenty residues apart (|i−j|≥20), but <8 Å away in terms of atomic distance between their alpha carbon ($C_{\alpha }$) atoms (Heffernan *et al.*, 2017). We computed the average number of non-local interactions per residue for each of the 1213 target proteins in the TEST2016 dataset and sorted the proteins in an ascending order of their average number of non-local interactions per residue. Next, we put them in six equal-sized bins ($b_{1},b_{2},\ldots ,b_{6}$) where the first bin contained the proteins with the lowest level of non-local interactions (0-0.61 non-local contacts per residue) and the sixth bin contains the proteins with the highest level of non-local interactions (1.64–2.70 non-local contacts per residue). Figure 3 shows the MAE(ϕ) and MAE(ϕ) for the best performing methods for these six bins.

These results show that—as expected—the performance of SAINT-Angle and other methods degrades as we increase the number of non-local contacts. However, SAINT-Angle is consistently and significantly (*P*-value ≪0.05) better than the best alternate methods across all levels of non-local interactions. Moreover, the improvements of SAINT-Angle (or SAINT-Angle-Single) over other methods tend to gradually increase with increasing levels of non-local interactions from $b_{1}$ to $b_{6}$ (with a few exceptions), especially for the torsion angle ψ.

Similarly, there is no notable difference between SPOT-1D and OPUS-TASS on $b_{1}$, whereas there are notable differences on $b_{6}$. These results indicate that long-range interactions have an impact on torsion angle prediction, and that capturing non-local interactions by self-attention modules is one of the contributing factors in the improvement of SAINT-Angle.

#### 3.6.2 Impact of 8-class (Q8) secondary structure states

We analyzed the performance of various methods on the 1213 target proteins in the TEST2016 dataset across eight types of secondary structure states, namely β-bridge (B), coil (C), β-strand (E), $3_{10}$-helix (G), α-helix (H), π-helix (I), bend (S) and β-turn (T). Supplementary Figure S3 in Supplementary Material shows the average MAE(ϕ) and MAE(ψ) against each of the Q8 labels for various methods. These results suggest that H (α-helix), G ($3_{10}$-helix), E (β-strand) and I (π-helix) regions usually have lower prediction errors whereas the non-ordinary states (Wang *et al.*, 2016), such as S (bend), C (coil), B (β-bridge) and T (β-turn) regions generally have higher prediction errors. Another notable observation is that SAINT-Angle consistently obtained superior performance across all Q8 labels compared to its competing methods, except that OPUS-TASS and ESIDEN obtained marginally better average MAE(ψ) values than SAINT-Angle on I states (see Supplementary Fig. S3b and d). Note that the I (π-helix) secondary state is extremely rare, appearing in only about 15% of all known protein structures, and is difficult to predict (Ludwiczak *et al.*, 2019). Notably, while the performances of different methods on the easy regions (e.g. H regions) are comparable, SAINT-Angle is notably better than other methods on regions where angle prediction is relatively hard (e.g. S and T regions).

### 3.7 Case study

**Fig. 4.**
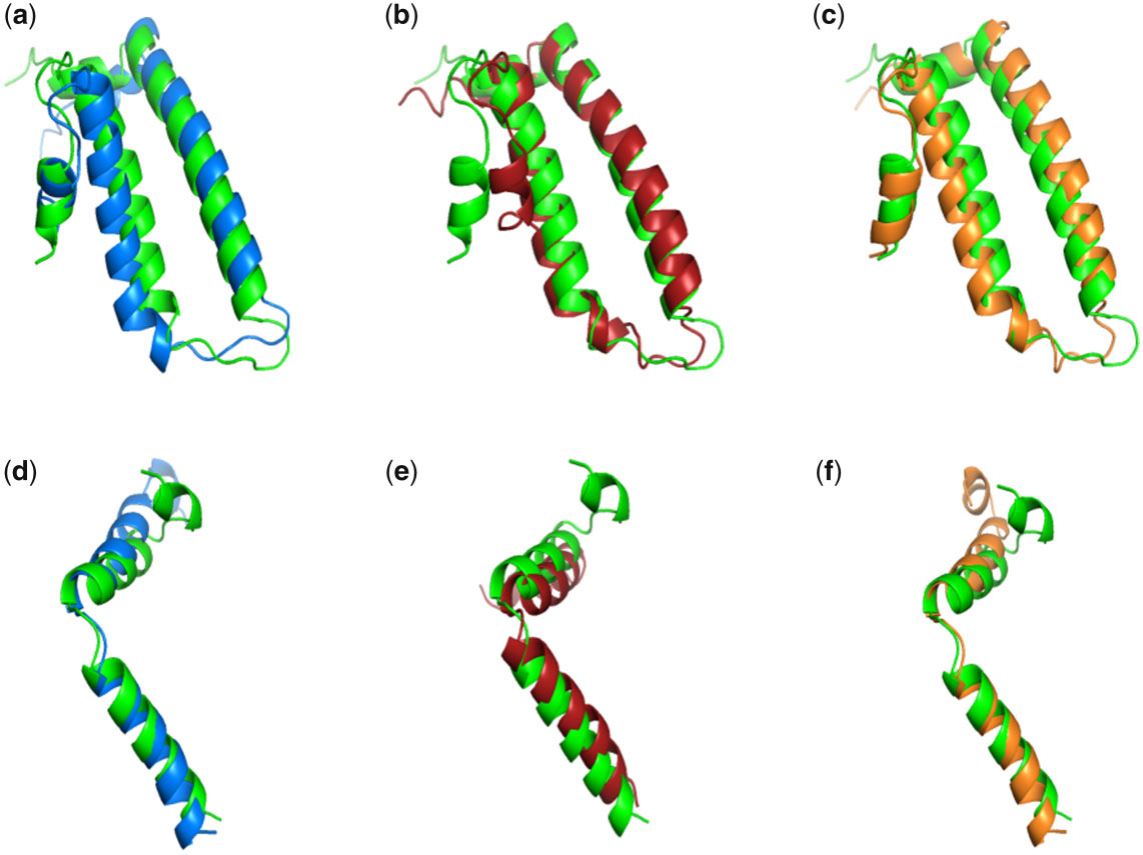
Superpositions of the structures of two representative proteins—5TDY (chain C) and 5LSI (chain D)—using the torsion angles estimated by SAINT-Angle, OPUS-TASS and ESIDEN with the structures using the native angles obtained from PDB. The structures with native angles are shown in green, and those with SAINT-Angle-Single, OPUS-TASS and ESIDEN predicted angles are shown in blue, red and orange, respectively. (**a–c**) Superpositions for the 5TDY (chain C) for SAINT-angle, OPUS-TASS and ESIDEN, respectively. (**d–f**) Superpositions for the 5LSI (chain D) protein. These images were made using PyMOL (Schrödinger, 2015)

In order to visually demonstrate the efficacy of SAINT-Angle in predicting the torsion angles, we conducted a case study to compare the protein backbone torsion angles predicted by SAINT-Angle-Single, ESIDEN and OPUS-TASS on two representative proteins from TEST2016 dataset (Hanson *et al.*, 2018), namely 5TDY (chain C) and 5LSI (chain D). For each method, we calculated the residue-wise absolute error (AE) of the predicted ϕ and ψ angles for these two proteins. We then plotted these residue-wise absolute errors against the corresponding secondary structure states (see Supplementary Table S9 and Supplementary Figs S1 and S2). These figures indicate that α-helix (H), $3_{10}$-helix (G) and bend (S) regions generally have lower prediction errors, while non-ordinary states such as coil (C) and β-turn (T) regions tend to have higher prediction errors. Our results show that SAINT-Angle-Single consistently provides better predictions across various secondary structure states compared to the other methods, especially for ψ angles. Figure 4 shows the predicted structures, superimposed on the native structures, of 5TDY (chain C) and 5LSI (chain D) proteins using the angles predicted by SAINT-Angle, OPUS-TASS and ESIDEN. The figure suggests that coiled and turn regions are not always in alignment with those regions in the native structures. However, helical and bend regions almost always bear similarities with those regions in the native structures.

### 3.8 Running time

Running time comparisons are presented in Supplementary Sect. 5 in the Supplementary Material.

## 4 Conclusions

We have presented SAINT-Angle, a highly accurate method for protein backbone torsion angles (ϕ and ψ) prediction. We have augmented the basic SAINT architecture for effective angle prediction and showed a successful utilization of transfer learning from pre-trained transformer-like language models. SAINT-Angle was assessed for its performance against the state-of-the-art backbone angles prediction methods on a collection of widely used benchmark datasets. Experimental results suggest that SAINT-Angle consistently improved upon the best existing methods.

The self-attention module in the SAINT architecture was particularly aimed for effectively capturing long-range interactions, and our systematic analyses of the performance of different methods under various model conditions with varying levels of long-range interactions indicate that SAINT-Angle can better handle complex models conditions with high levels of long-range interactions. The improvement of SAINT-Angle over other methods in ψ prediction, which is typically harder to predict than ϕ, is noteworthy as it achieved more than 2–6 degree less MAE(ψ) than other methods on benchmark datasets.

We utilized transfer learning using the extracted features from the protein language model ProtTrans . The *ProtTrans* architecture (discussed in Sect. 2.2.2.2) alone (i.e. without the ensemble network) performs better than the existing best methods like OPUS-TASS and SPOT-1D—showing the positive impact of transfer learning in protein attribute prediction. We also analyzed the novel evolutionary features proposed in Xu *et al.* (2021). Our analyses with the evolutionary features reconfirms the effectiveness of the evolutionary features in protein angle prediction which was first demonstrated by Xu *et al.* (2021). Our results also suggest that the architecture of SAINT-Angle is *feature-robust*, as it performs well with different types of features [e.g. ProtTrans features, evolutionary features proposed by Xu *et al.* (2021)] and consistently outperforms other competing methods on varying feature sets. Given this demonstrated performance improvement on various benchmark datasets and under challenging model conditions, we believe SAINT-Angle advances the state-of-the-art in this domain, and will be considered as a useful tool for predicting the backbone torsion angles.

This study can be extended in several directions. As an immediate future direction, we plan to train a multi-task learning model by leveraging the original SAINT and the proposed SAINT-Angle architectures, which will simultaneously predict residue-wise protein secondary structures along with backbone torsion angles. Although SAINT-Angle appears to be feature-robust, follow-up studies need to investigate various features and select a small set of simple features that are sufficient for SAINT-Angle to predict backbone angles with reasonable accuracy. Our study suggests that SAINT-Angle—even when run with only the ProtTrans features (i.e. without using any alignment profile-based features such as PSSM or HMM)—performs notably well. Therefore, pretrained protein language model-based methods do not appear to require a certain level of homologous sequences for reasonably accurate prediction. On the other hand, MSA-based methods are less successful on ‘orphan proteins’—proteins with few or no homologs (Chowdhury *et al.*, 2022; Michaud *et al.*, 2022). For example, AlphaFold2 (Jumper *et al.*, 2021)—which made remarkable advancements in protein structure prediction accuracy—requires at least 30 effective homologous sequences to achieve an accurate structure prediction (Singh et al., 2022b). Chowdhury *et al.* (2022) presented a protein language model (AminoBERT)-based method which outperformed AlphaFold2 in predicting structures of orphan proteins. However, further extensive studies are necessary to draw firm conclusions on how the performance of language model-based methods vary with varying amounts of homologous sequences. Another future research direction is to extend and utilize the proposed model as well as predicted angles to infer other protein attributes. This may allow us to observe the effect of the improvement, our method brings about, in downstream analyses.

## Supplementary Material

vbad042_Supplementary_Data

## Data Availability

The software and data are available at https://github.com/bayzidlab/SAINT-Angle.
