# Supplementary material for: SAINT-Angle: self-attention augmented inception-inside-inception network and transfer learning improve protein backbone torsion angle prediction
Source: Bioinform Adv. 2023 Apr 5;3(1):vbad042. doi: 10.1093/bioadv/vbad042 (PMC10115468; doi:10.1093/bioadv/vbad042)
Supplement: vbad042_Supplementary_Data [file vbad042_supplementary_data.pdf]

Supplementary Materials to SAINT-Angle: self-attention augmented inception-inside-inception network and transfer learning improve protein backbone torsion angle prediction

A.K.M. Mehedi Hasan[1,†], Ajmain Yasar Ahmed[1,†], Sazan Mahbub[1,2], M. Saifur Rahman[1], and Md. Shamsuzzoha Bayzid[1,*]

[1]Department of Computer Science and Engineering
Bangladesh University of Engineering and Technology
Dhaka-1205, Bangladesh
[2]Department of Computer Science
University of Maryland
College Park, Maryland 20742, USA
[†]These authors contributed equally to this work.
[*]Corresponding author: shams_bayzid@cse.buet.ac.bd

1 Overview

These supplementary materials present additional details about the base models used in our ensemble network (Sec. 2), ablations studies (Sec. 3), the datasets used to validate and test our models (Sec. 4), running time analysis (Sec. 5), torsion angle prediction case study (Sec. 6), and additional analysis of predicted angles (Sec. 7).

2 Base Models in Ensemble Network

We initially trained 24 different models with different feature sets and model architectures. The same training and validation sets that were used by SPOT-1D [Hanson et al., 2019] were used to train these models and tune necessary hyperparameters. Different combinations of model architectures and feature sets were explored. In addition to the base, ProtTrans, and window features (as discussed in Sec. 2.1 in the main paper), we made an attempt to use the native 8-class (Q8) secondary structure (SS) of the residues as a feature, which resulted in a significant improvement in backbone angle prediction (results not shown). However, native structure is not available for all proteins, especially for the newly discovered ones. So, we used SAINT [Uddin et al., 2020] to produce predicted probability distribution of the Q8 states of the residues in a given protein. We call this predicted Q8 states the *predicted features*. Thus, we used 16 different feature sets as shown in Table S1. As we will show later, unlike the true SS, using the predicted features did not result in notable improvements in backbone angle prediction.

We used the *Basic* architecture on feature sets $fs1 - fs8$, thereby producing eight models. On features sets $fs9-fs16$, we applied both the *ProtTrans* and *Residual* architectures, resulting in 16 different models. Thus, in total, we trained 24 models. Hence, we obtained 24 models in total. We evaluated the performance of each of these models on the validation set (see

Table S1: Different feature sets and their acronyms.

Feature set	Notation	Feature set	Notation
Base	fs1	Base+ProtTrans	fs9
Base+Win10	fs2	Base+ProtTrans+Win10	fs10
Base+Win20	fs3	Base+ProtTrans+Win20	fs11
Base+Win50	fs4	Base+ProtTrans+Win50	fs12
Base+Predicted	fs5	Base+ProtTrans+Predicted	fs13
Base+Win10+Predicted	fs6	Base+ProtTrans+Win10+Predicted	fs14
Base+Win20+Predicted	fs7	Base+ProtTrans+Win20+Predicted	fs15
Base+Win50+Predicted	fs8	Base+ProtTrans+Win50+Predicted	fs16

Table S2). Besides, we conducted an inclusion-exclusion study to select a set of models for an ensemble network. We did not observe any advantage of using the predicted features. Finally, we selected 8 models, as shown in Table S3 to form the ensemble model which we call SAINT-Angle. Table S4 shows the comparison of these based models with existing best performing methods on TEST2016 and TEST2018 benchmark datasets.

Table S2: Performance of the 24 individual models on the validation set.

Feature set	Basic architecture		Feature	ProtTrans Architecture		Residual Architecture	
	MAE(ϕ)	MAE(ψ)		MAE(ϕ)	MAE(ψ)	MAE(ϕ)	MAE(ψ)
fs1	16.77	23.69	fs9	15.79	20.95	15.73	21.23
fs2	16.38	22.74	fs10	15.63	20.67	15.56	20.67
fs3	16.35	22.79	fs11	15.64	20.75	15.61	20.80
fs4	16.41	22.80	fs12	15.69	20.74	15.75	20.94
fs5	16.43	22.85	fs13	15.70	20.90	15.63	20.83
fs6	16.40	22.89	fs14	15.68	21.05	15.61	20.95
fs7	16.44	22.85	fs15	15.72	20.97	15.61	21.05
fs8	16.43	22.86	fs16	15.71	21.09	15.68	21.04

Table S3: Performance of the selected eight base models and the corresponding ensemble network on the validation set.

Model	Feature set	Architecture	validation error	
			MAE(ϕ)	MAE(ψ)
Model 0	fs1	Basic	16.77	23.69
Model 1	fs2	Basic	16.38	22.74
Model 2	fs9	ProtTrans	15.79	20.95
Model 3	fs10	ProtTrans	15.63	20.67
Model 4	fs11	ProtTrans	15.64	20.75
Model 5	fs12	ProtTrans	15.69	20.74
Model 6	fs9	Residual	15.73	21.23
Model 7	fs10	Residual	15.56	20.67
Ensemble			15.39	20.14

Table S4: Performance (in terms of MAE(ϕ) and MAE(ψ)) of the individual base models used in SAINT-Angle and the best competing methods on TEST2016 and TEST2018. The best and the second best results are shown in bold and italic, respectively.

Method	TEST2016		TEST2018	
	MAE(ϕ)	MAE(ψ)	MAE(ϕ)	MAE(ψ)
Model 0	16.80	24.38	17.37	25.93
Model 1	16.51	23.70	16.99	25.20
Model 2	15.95	21.88	16.18	23.04
Model 3	*15.76*	**21.45**	16.16	**22.71**
Model 4	15.79	*21.50*	*16.13*	*22.76*
Model 5	15.84	21.54	16.21	22.78
Model 6	15.91	22.02	16.58	23.47
Model 7	**15.75**	21.57	16.58	23.06
SPOT-1D[a]	16.27	23.26	16.89	24.87
OPUS-TASS[b]	15.78	22.46	16.40	24.06
SPOT-1D-LM[c]	-	-	**15.99**	23.74

[a]Results reported by SPOT-1D [Hanson et al., 2019].

[b]Results reported by OPUS-TASS [Xu et al., 2020].

[c]Results reported by SPOT-1D-LM [Singh et al., 2022].

2.1 Ensemble using the features proposed by ESIDEN

In order to compare with ESIDEN [Xu et al., 2021] and to take advantage of the novel features proposed by ESIDEN, we removed the window features and used the ESIDEN features instead – resulting in three models as shown in Table S5. So, in this case, the feature set contains 3 basic features including 20 types of amino acids (AA), 7 physicochemical properties (PCP), and position-specific scoring matrix (PSSM) along with 4 novel evolutionary features proposed by ESIDEN, namely degree of conservation (DC), relative entropy (RE), position-specific substitution probabilities (PSSP), and Ramachandran basin potential (RBP). We call this variant, which is an ensemble of the three models as shown in Table S5, SAINT-Angle*.

Table S5: Three models used in SAINT-Angle* when the ESIDEN features are available. We show the architectures and features used in these three models.

Model	Architecture	Feature set
0	Basic	Base (PSSM, HMM, PCP) + ESIDEN (AA, DC, RE, PSSP, RBP)
1	ProtTrans	Base+ESIDEN+ProtTrans
2	Residual	Base+ESIDEN+ProtTrans

3 Impact of different features and model architectures used in SAINT-Angle

We conducted ablation studies to evaluate the impact of different feature sets and model architectures on our proposed method. We carried out two evaluations to demonstrate the performance of our method on the validation set using different feature sets and different model architectures, respectively. These results further supported our choice of base models for ensemble learning as well as the reasoning behind designing and using three different architectures.

Table S6: Performance of the ProtTrans architecture on different feature sets on the validation dataset.

Feature set	ProtTrans Architecture	
	MAE(ϕ)	MAE(ψ)
fs1	16.98	23.87
fs2	16.56	22.73
fs3	16.54	22.70
fs4	16.61	22.80
ProtTrans[a]	16.42	22.66
fs9	15.79	20.95
fs10	15.63	20.67
fs11	15.64	20.75
fs12	15.69	20.74

[a]Using ProtTrans features only.

Table S6 shows the contributions of different feature sets to prediction of protein backbone torsion angles ϕ and ψ. We conducted this analysis using *ProtTrans* architecture on feature sets fs1-fs4 and fs9-fs12. Additionally, we tested the performance of this model architecture with only ProtTrans features. As shown in Table S6, the prediction performance of SAINT-Angle was improved with the inclusion of different Window features and significantly boosted with the inclusion of ProtTrans features. The analysis also shows the strength of extracted features from pre-trained protein language models [Elnaggar et al., 2020] in torsion angle prediction. SAINT-Angle with only ProtTrans features performed better than that with Base and Window features. Nevertheless, as shown in Tables S2 and S6, the base model with the *Basic* architecture trained on only biological (Basic and Window) features generated better prediction results than the ProtTrans architecture trained on only Basic and Window features. This analysis backs our decision to choose the base models listed in Table S3 for ensembling.

Table S7: Performance of different architectures on both base and ProtTrans features on the validation dataset.

Feature set	Basic Architecture		ProtTrans Architecture		Residual Architecture	
	MAE(ϕ)	MAE(ψ)	MAE(ϕ)	MAE(ψ)	MAE(ϕ)	MAE(ψ)
Base (fs1)	16.77	23.69	16.98	23.87	19.54	25.91
ProtTrans[a]	16.62	22.80	16.42	22.66	16.40	22.52

[a]Using ProtTrans features only.

Table S7 presents the impact of various model architectures on the prediction of backbone torsion angles. The analysis was conducted using the Basic, ProtTrans, and Residual architectures, with both Base features (fs1) and ProtTrans features. As shown in Table S7, the Basic architecture performed best when we used the Base features. However, the use of ProtTrans and Residual architectures improved the prediction performance when ProtTrans features were incorporated. This analysis supports our decision to use the Basic architecture with biological features and the ProtTrans and Residual architectures with the extracted features from the protein language model. This indicates that we effectively customized our ProtTrans architecture specifically to leverage the ProtTrans features and the Basic architecture to make the most of the basic features.

4 Test Datasets

TEST2016 TEST2016 was compiled by [Hanson et al., 2018], containing 1213 proteins that were deposited on PDB [Berman et al., 2000] between June 2015 and February 2017 with similar parameter settings as the training and validation sets. None of the proteins contains more than 700 amino acid residues. The dataset has <25% sequence similarity with the training and validation sets according to BlastClust.

TEST2018 TEST2018 dataset, compiled by [Hanson et al., 2019], contains 250 high-quality, non-redundant proteins that were deposited in PDB between January 2018 and July 2018 with the similar parameter settings and filtering constraints as the TEST2016 dataset.

TEST2020-HQ TEST2020-HQ dataset, compiled by [Singh et al., 2022], is a hard test set including 124 proteins, all of which were released between May 2018 and April 2020. This dataset was obtained by removing close and remote homologs and with similar parameter settings as well as filtering constraints as the TEST2018 dataset. Proteins consisting of over 700 amino acid residues were removed for efficient calculations [Hanson et al., 2018]. As a result, 121 proteins remained in the TEST2020-HQ dataset.

CAMEO109 CAMEO109 dataset contains 109 proteins that were released between March 2021 and June 2021 by the community project CAMEO (Continuous Automated Model Evaluation) [Haas et al., 2013]. These proteins contains less than 500 amino acid residues and have <25% sequence similarity with the SPOT-1D training set according to BlastClust.

CASP We used a collection of CASP [Koehl and Levitt, 1999] (Critical Assessment of protein Structure Prediction) datasets, especially the recent ones (e.g., CASP12 and CASP13). We used another dataset CASP-FM, comprising the CASP Free Modeling (FM) targets, which was previously compiled by the authors of SAINT. CASP-FM contains 56 domain sequences: 10 FM targets from CASP13, 22 FM targets from CASP12, 16 FM targets (out of 30) from CASP11 and 8 FM targets (out of 12) from CASP10.

5 Running time

We compared the computational speed of ESIDEN [Xu et al., 2021] and SAINT-Angle-Single, both trained on features from ESIDEN, by analyzing their inference time on the TEST2016 and TEST2018 datasets. The running times, as shown in Table S8, demonstrate that SAINT-Angle-Single is notably faster than ESIDEN. This evaluation was performed on a device with 64GB RAM and an NVIDIA RTX3070Ti GPU (8GB VRAM), taking into account only the prediction time.

We were unable to run other competing methods either because they were not publicly accessible or challenging to configure on our local machines. The authors of the OPUS-TASS paper [Xu et al., 2020] reported a comparison of computational speed between OPUS-TASS and SPOT-1D [Hanson et al., 2019] on the TEST2018 dataset. SAINT-Angle performed significantly faster, with 44 sec of inference time, than these two methods which took 150 sec and 678 sec respectively. Remarkably, SAINT-Angle-Single took only 1.22 sec of inference time on this dataset and achieved better performance than other methods. Although it clearly shows that SAINT-Angle is faster than the competing methods, the difference in device configurations (especially, GPU), used to conduct the analyses in OPUS-TASS study, might partly contribute to this notable difference in computational speed.

Table S8: Computational speed analysis between ESIDEN and SAINT-Angle on TEST2016 and TEST2018 datasets. We show the time required by these two methods to predict the torsion angles of the proteins in these two datasets.

Dataset	Method	
	ESIDEN	SAINT-Angle
TEST2016	12.01 sec	9.11 sec
TEST2018	2.27 sec	1.22 sec

6 Case Study

We conducted a case study to compare the protein backbone torsion angles ϕ and ψ prediction performance of SAINT-Angle-Single with ESIDEN [Xu et al., 2021] and OPUS-TASS [Xu et al., 2020] on two representative proteins. For this particular case study, we used the SAINT-Angle-Single model with the *Basic* architecture, trained on ESIDEN features.

Table S9: Mean absolute errors (MAE) of different methods on 5TDY (Chain C) and 5LSI (Chain D).

Protein	SAINT-Angle		ESIDEN		OPUS-TASS	
	MAE(ϕ)	MAE(ψ)	MAE(ϕ)	MAE(ψ)	MAE(ϕ)	MAE(ψ)
5TDY (Chain C)	21.06	19.87	23.83	22.39	24.91	38.50
5LSI (Chain D)	15.21	17.90	17.47	23.69	18.21	26.31

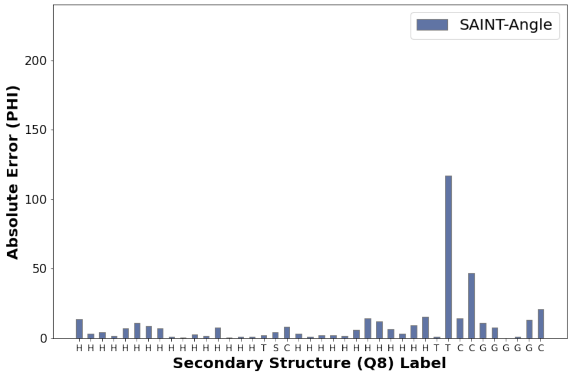

(a)

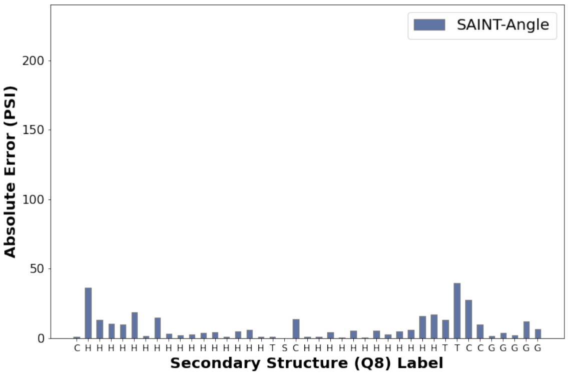

(b)

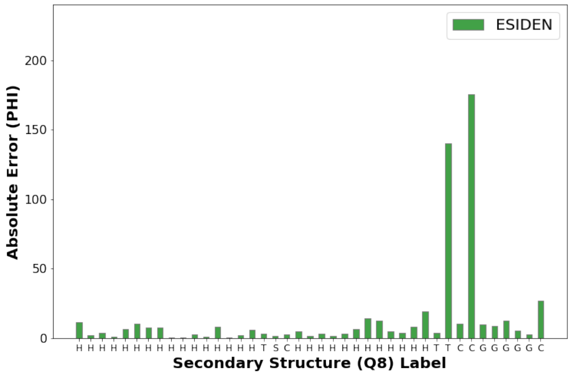

(c)

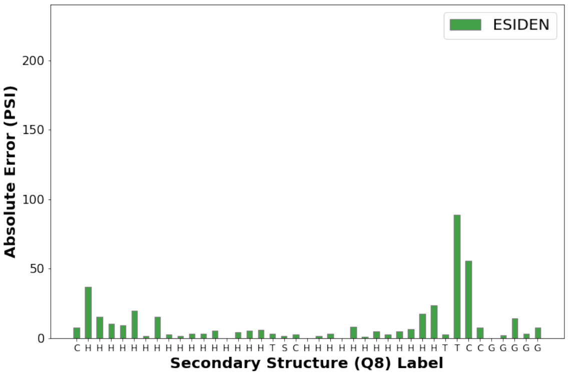

(d)

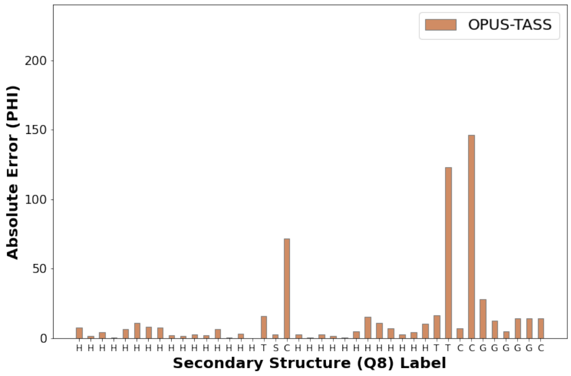

(e)

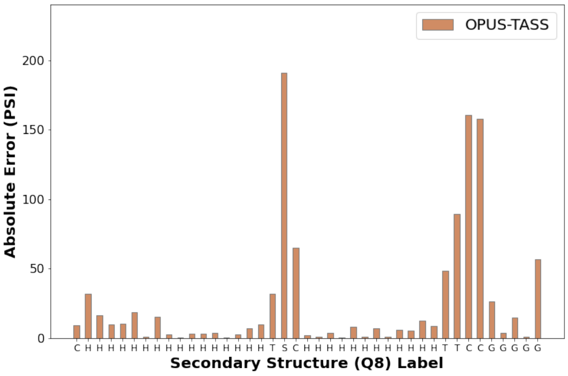

(f)

Figure S1: Residue-wise absolute error (AE) of different methods on dihedral ϕ and ψ angles prediction for 5TDY (chain C) protein. We plot absolute errors against the Q8 secondary structures of the protein. **(a)-(b)** AE(ϕ) and AE(ψ) for SAINT-Angle. **(c)-(d)** AE(ϕ) and AE(ψ) for ESIDEN. **(e)-(f)** AE(ϕ) and AE(ψ) for OPUS-TASS.

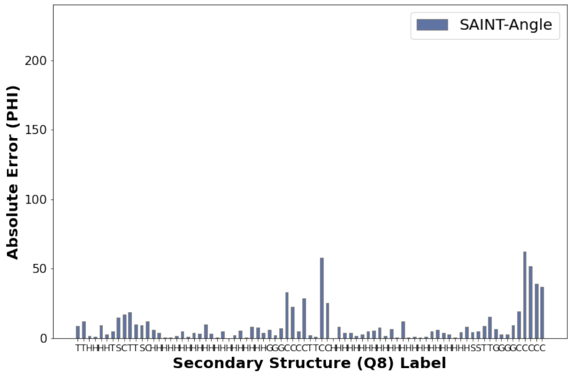

(a)

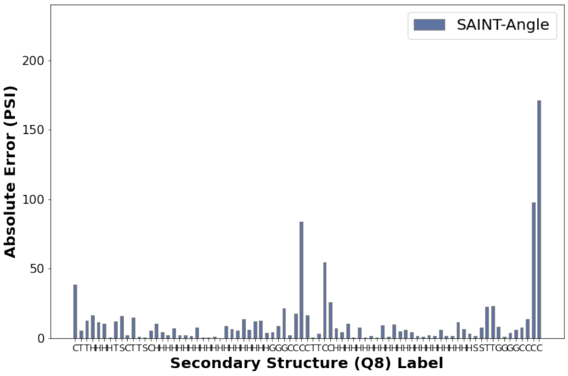

(b)

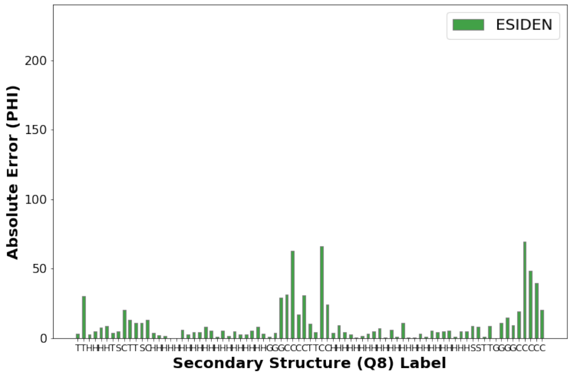

(c)

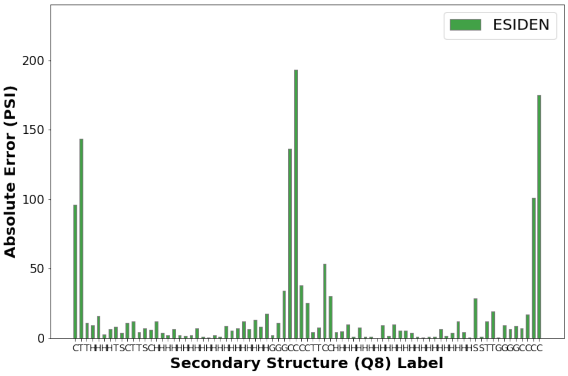

(d)

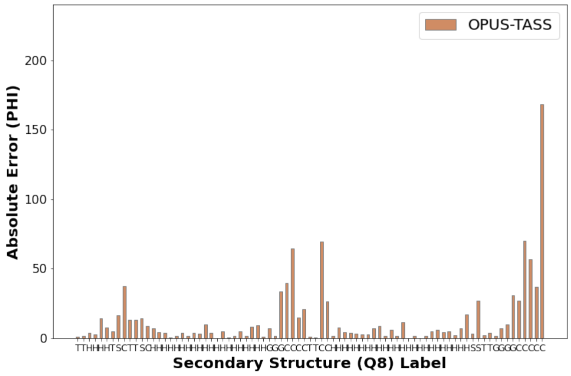

(e)

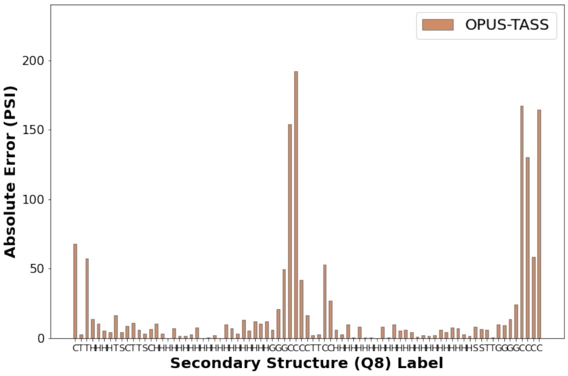

(f)

Figure S2: Residue-wise absolute error (AE) of different methods on dihedral ϕ and ψ angles prediction for 5LSI (chain D) protein. **(a)-(b)** AE(ϕ) and AE(ψ) for SAINT-Angle. **(c)-(d)** AE(ϕ) and AE(ψ) for ESIDEN. **(e)-(f)** AE(ϕ) and AE(ψ) for OPUS-TASS.

7 Additional Analysis of Predicted Angles

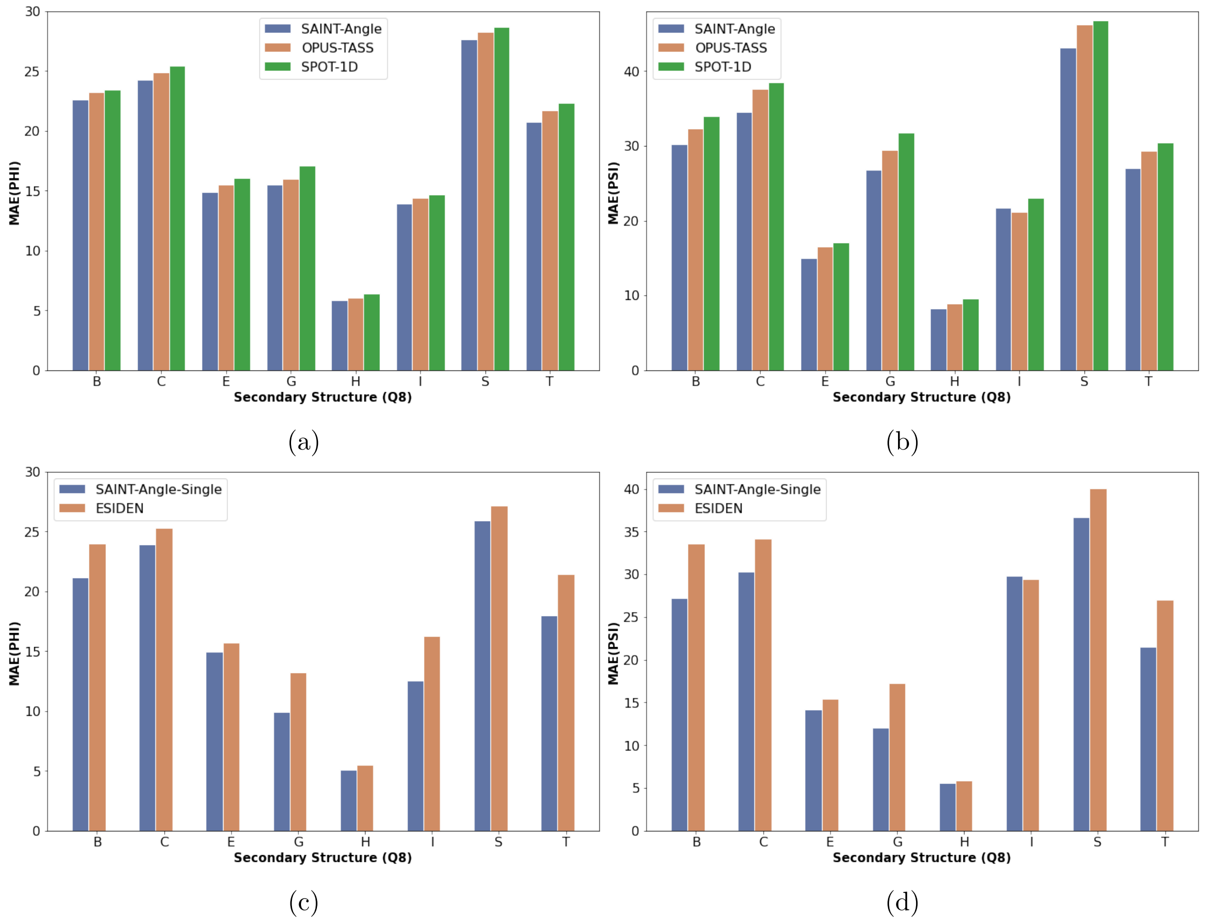

Figure S3: MAE(ϕ) and MAE(ϕ) of different methods on 8-class secondary structures for proteins in the TEST2016 dataset [Hanson et al., 2018]. **(a)-(b)** MAE(ϕ) and MAE(ψ) of SAINT-Angle, OPUS-TASS, and SPOT-1D across different Q8 structures. **(c)-(d)** MAE(ϕ) and MAE(ψ) of SAINT-Angle-Single and ESIDEN across different Q8 structures.

References

H. M. Berman, J. Westbrook, Z. Feng, G. Gilliland, T. N. Bhat, H. Weissig, I. N. Shindyalov, and P. E. Bourne. The protein data bank. *Nucleic acids research*, 28(1):235–242, 2000.

A. Elnaggar, M. Heinzinger, C. Dallago, G. Rihawi, Y. Wang, L. Jones, T. Gibbs, T. Feher, C. Angerer, D. Bhowmik, et al. Prottrans: Towards cracking the language of life's code through self-supervised deep learning and high performance computing. *arXiv preprint arXiv:2007.06225*, 2020.

J. Haas, S. Roth, K. Arnold, F. Kiefer, T. Schmidt, L. Bordoli, and T. Schwede. The protein model portal—a comprehensive resource for protein structure and model information. *Database*, 2013, 2013.

J. Hanson, K. Paliwal, T. Litfin, Y. Yang, and Y. Zhou. Accurate prediction of protein con-

tact maps by coupling residual two-dimensional bidirectional long short-term memory with convolutional neural networks. *Bioinformatics*, 34(23):4039–4045, 2018.

J. Hanson, K. Paliwal, T. Litfin, Y. Yang, and Y. Zhou. Improving prediction of protein secondary structure, backbone angles, solvent accessibility and contact numbers by using predicted contact maps and an ensemble of recurrent and residual convolutional neural networks. *Bioinformatics*, 35(14):2403–2410, 2019.

P. Koehl and M. Levitt. A brighter future for protein structure prediction. *nature structural biology*, 6(2):108–111, 1999.

J. Singh, K. Paliwal, T. Litfin, J. Singh, and Y. Zhou. Reaching alignment-profile-based accuracy in predicting protein secondary and tertiary structural properties without alignment. *Scientific Reports*, 12(1):7607, May 2022. ISSN 2045-2322. doi: 10.1038/s41598-022-11684-w. URL https://doi.org/10.1038/s41598-022-11684-w.

M. R. Uddin, S. Mahbub, M. S. Rahman, and M. S. Bayzid. SAINT: self-attention augmented inception-inside-inception network improves protein secondary structure prediction. *Bioinformatics*, 36(17):4599–4608, 2020.

G. Xu, Q. Wang, and J. Ma. Opus-tass: a protein backbone torsion angles and secondary structure predictor based on ensemble neural networks. *Bioinformatics*, 36(20):5021–5026, 2020.

Y.-C. Xu, T.-J. ShangGuan, X.-M. Ding, and N. J. Cheung. Accurate prediction of protein torsion angles using evolutionary signatures and recurrent neural network. *Scientific reports*, 11(1):1–11, 2021.
